# Impact of the novel coronavirus infection on pediatric surgery: an analysis of data from the National Clinical Database

**DOI:** 10.1007/s00595-024-02792-3

**Published:** 2024-02-13

**Authors:** Kazuya Ise, Hisateru Tachimori, Jun Fujishiro, Hirofumi Tomita, Kan Suzuki, Hiroyuki Yamamoto, Hiroaki Miyata, Yasushi Fuchimoto

**Affiliations:** 1 NCD Liaison Committee of the Japanese Society of Pediatric Surgeons, Tokyo, Japan; 2 Department of Pediatric Surgery, Yamagata Prefectural Central Hospital, Yamagata, Yamagata 990-2292 Japan; 3 Endowed Course for Health System Innovation, Keio University School of Medicine, Tokyo, Japan; 4 Department of Healthcare Quality Assessment, Graduate School of Medicine, The University of Tokyo, Tokyo, Japan; 5 Department of Pediatric Surgery, The University of Tokyo, Tokyo, Japan; 6 Department of Surgery, Tokyo Metropolitan Children’s Medical Center, Tokyo, Japan; 7 Surgical Oncology Graduate School of Medicine, Dokkyo Medical University, Mibu, Japan; 8 Department of Pediatric Surgery, International University of Health and Welfare, Narita, Japan

**Keywords:** COVID-19, Pediatric surgery, National Clinical Database, Japan

## Abstract

**Purpose:**

The coronavirus disease 2019 (COVID-19) pandemic limited the delivery of medical resources. Although surgeries are triaged according to disease severity and urgency, a delay in diagnosis and surgery can be detrimental. We conducted this study to analyze data on the impact of the COVID-19 pandemic on pediatric surgery for different diseases or disorders.

**Methods:**

We compiled and compared data on pediatric surgical cases from 2018 to 2020, using the National Clinical Database. The number of diseases, severity, complication rates, mortality rates by disease/disorder, and the COVID-19 pandemic areas were analyzed.

**Results:**

The total number of cases of pediatric surgery in 2018, 2019, and 2020 was 50,026, 49,794, and 45,621, respectively, reflecting an 8.8% decrease in 2020 from 2018 and an 8.4% decrease in 2020 from 2019. A decrease was observed when the number of patients with COVID-19 was high and was greater in areas with a low infection rate. There was a marked decrease in the number of inguinal hernia cases. The number of emergency room visits and emergency surgeries decreased, but their relative proportions increased.

**Conclusions:**

The COVID-19 pandemic decreased the number of pediatric surgeries, reflecting the limitations of scheduled surgeries and infection control measures.

## Introduction

In 2020, a worldwide coronavirus disease 2019 (COVID-19) pandemic occurred, caused by severe acute respiratory syndrome coronavirus 2 [1]. The necessity to curtail elective surgeries during this period from the perspective of infection control and resource conservation caused significant disruption to health care systems around the world [2, 3]. Surgeries for elective, urgent, and emergency cases were delayed significantly, exacerbating existing surgical backlogs [4]. A lack of operating room space and hospital beds were major barriers to the provision of surgical treatment, and long surgical waiting times impacted access to surgery. Now is the time to utilize limited surgical resources and develop strategies and waiting lists for future pandemics [4]. The number of infected individuals throughout Japan increased with three epidemic waves in 2020 [5]. The spread of infections and changes in the medical environment changed social and family life, along with the worsening of medical conditions and increased mortality because of delayed diagnosis caused by limited opportunities for consultation and medical care. Surgery is performed for various pediatric diseases and disorders; however, as the disease severity and timing of surgery vary and the number of patients with similar conditions decreases, simple quantitative comparisons are difficult. This study examines the impact of the COVID-19 pandemic on pediatric surgical practice based on data from the National Clinical Database (NCD) registry, which covers surgical care nationwide. We focused on the number of pediatric surgeries at the regional infection level, surgical triage, severity of specific diseases, surgical type, emergency situations, mortality and morbidity, and chronological changes in the number of operations, including their correlation with surgical complexity, whether advanced or not.

## Methods

### Data collection

Data from the NCD, which registered treatments performed nationwide from 2018 to 2020 for patients younger than 16 years of age at the time of surgery, were used for this study, which included neonatal non-surgical cases. Differences between the 2018 and 2019 data were considered an annual change and compared with the data from 2020 to establish whether the COVID-19 pandemic had an impact on pediatric surgical practice. The total number of registered cases and data from the Annual Report 2019 [6] were tabulated to determine the change in the number of instances by procedure and disease specificity, which included 20 diseases or disorders with at least 100 cases per year. We analyzed and compared the number and proportion of cases by month, week, and region, as well as the number of infected people, disease severity, unexpected reoperations, readmissions, and surgical deaths.

### Target procedure

Overall, 20 diseases or disorders with more than 100 cases per year were included in the disease-specific survey [6]. Surgeries for these 20 diseases or disorders included the following: inguinal hernia repair; appendectomy; orchidopexy; gastrostomy; gastrointestinal obstruction surgery; colostomy closure; gastrointestinal perforation surgery; fundoplication; lung resection; gastrointestinal atresia surgery; malignant tumor radical surgery; malrotation surgery; Hirschsprung’s disease radical surgery; congenital biliary dilatation surgery; diaphragmatic hernia repair; excision of mediastinal, retroperitoneal, and presacral benign tumors; funnel chest surgery; high–mid-level anorectal malformation radical surgery; low-level anorectal malformation radical surgery; and primary repair of gastroschisis. The highly advanced surgery group was selected based on surgeries approved by the Pediatric Surgery Specialty Program. Highly advanced surgeries included the following (registered in the list): gastrointestinal perforation surgery, gastrointestinal obstruction surgery, fundoplication, anorectal malformation radical surgery, malignant tumor radical surgery, lung resection, funnel chest surgery, Hirschsprung’s disease radical surgery, biliary atresia surgery, congenital biliary dilatation surgery, malrotation surgery, tracheal stenosis surgery, esophagectomy, esophageal reconstruction surgery, gastrointestinal atresia surgery, diaphragmatic hernia repair, primary repair of gastroschisis, hepatectomy, portal hypertension surgery, excision of mediastinal tumors (retroperitoneal and presacral benign tumors), ectopic bladder/ectopic sulcus surgery, pancreatectomy, urethroplasty, vesicoureteral reflux surgery, urinary tract alteration surgery, nephrectomy, ureterectomy, pelvic urethroplasty, bladder enlargement, female genital surgery, liver transplantation, small intestine transplantation, kidney transplantation, total colectomy (except for malignant diseases), laryngotracheal separation, splenectomy, and colostomy closure.

### Surgical triage

Pediatric surgical disease levels set by the Japanese Association of Pediatric Surgeons were established based on the guidelines for surgical triage in the Elective Surgery Acuity Scale of Saint Louis University, as recommended by the American College of Surgeons [7]. Disease level A was defined as non-fatal or non-urgent surgery (inguinal hernia repair, orchidopexy, gastrostomy, colostomy closure, fundoplication, funnel chest surgery, Hirschsprung’s disease radical surgery, and excision of mediastinal, retroperitoneal, and presacral benign tumors). Disease level B was defined as non-fatal but potentially life-threatening or critical surgery (gastrointestinal obstruction surgery, high–mid-level anorectal malformation radical surgery, congenital biliary dilatation surgery, and lung resection). Disease level C was defined as surgery for a disease that is potentially fatal, if not performed within days to months (appendectomy, gastrointestinal perforation surgery, malignant tumor radical surgery, low-level anorectal malformation radical surgery, gastrointestinal atresia surgery, malrotation surgery, diaphragmatic hernia repair, and primary repair of gastroschisis).

### Classification of prefectures according to the degree of infection

Based on the cumulative number of infected persons/population by prefecture (December 31, 2020), regions were divided into three categories: high, medium, and low (the top-, bottom-, and middle-ranked regions were not included in any category) [8]. There were 12 prefectures with a high number of infections: Aichi, Chiba, Fukuoka, Hokkaido, Hyogo, Kanagawa, Kyoto, Nara, Okinawa, Osaka, Saitama, and Tokyo; 22 prefectures with a medium number of infections: Fukushima, Gifu, Gunma, Hiroshima, Ibaraki, Ishikawa, Kagoshima, Kochi, Kumamoto, Mie, Miyagi, Miyazaki, Nagano, Oita, Okayama, Saga, Shiga, Shizuoka, Tochigi, Toyama, Yamanashi, and Wakayama; and 13 prefectures with a low number of infections: Akita, Aomori, Ehime, Fukui, Iwate, Kagawa, Nagasaki, Niigata, Shimane, Tokushima, Tottori, Yamagata, and Yamaguchi.

### Data evaluation

We examined the monthly changes in the total number of registered cases, surgeries performed for the 20 diseases or disorders, and advanced surgeries, as well as the weekly changes in inguinal hernia repair, which is a common procedure. We also evaluated the severity of appendicitis, which may be progressive if treatment is withheld. Emergency transport, emergency surgery, postoperative outcomes, and deaths were compared.

### Statistical analysis

To evaluate the severity of the four significant diseases, emergency transport, emergency surgery, postoperative outcomes, and deaths, an *χ*^2^ test was performed to examine differences in the number of surgeries per year. The number of surgeries per year and corresponding 95% confidence intervals (CIs) are shown by endemicity and the type of surgery. Monthly or weekly surgical procedures were examined using the following methods to establish whether the number of surgeries during the pandemic period fell within the range predicted by the actual number of surgeries performed prior to the pandemic period. For the pandemic period, we estimated the parameters of an ARIMA model with seasonality, using the data obtained from the pre-pandemic period. For this estimation, the auto.arima function of the forecast package was used, and the best model was selected as an indicator. Using this model, we obtained the predicted values and their intervals (70% and 95%) for the post-pandemic period, which were plotted in time-series plots overlaid with the observed values. On January, 30, 2020, the World Health Organization (WHO) declared a Public Health Emergency of International Concern for the novel coronavirus infection. Subsequently, on March 11, based on the status of the global spread of the disease and the severity of the outbreak, the WHO declared the new coronavirus infection a pandemic. In this study, the post-pandemic period is defined as March, 2020 or later for monthly data, and week 13 or later for weekly data. All statistical analyses were performed using R version 4.0 or later (R Core Team, 2023) [9]. All tests were two-tailed and the significance level was set at 0.05.

### Ethical concerns

This study was performed in accordance with the ethical standards laid down in the 1964 Declaration of Helsinki and its later amendments and was approved by the Ethics Review Committee (approval number 2021–10) of the Japanese Red Cross Sendai Red Cross Hospital and by the board of the Japanese Society of Pediatric Surgeons as not subject to review (approval Sun 2021/11/18). No written consent was obtained from the patients because the data excluded personal information. An opt-out form was posted on the NCD website as a request for cooperation in academic research of NCD data.

## Results

**Fig. 1:**
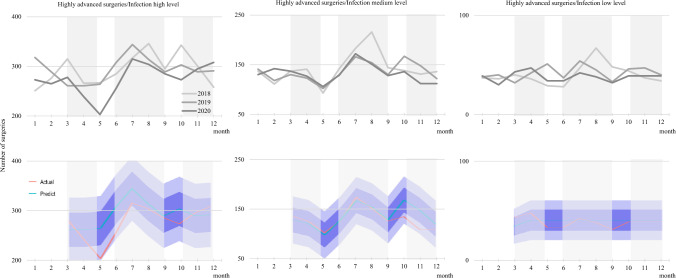
Time-series analysis of monthly changes in the number of difficult surgeries by region, divided into three prefectures according to the infection level (2018–2020): **a** highly difficult surgeries: high infection level; **b** highly difficult surgeries: medium infection level; **c** highly difficult surgeries: low infection level. A model was created to predict the number of surgeries based on pre-pandemic data, and the number of post-pandemic surgeries predicted by the model was plotted over the actual number of surgeries. The 70% and 95% confidence intervals for the predicted number of surgeries are also displayed

**Fig. 2:**
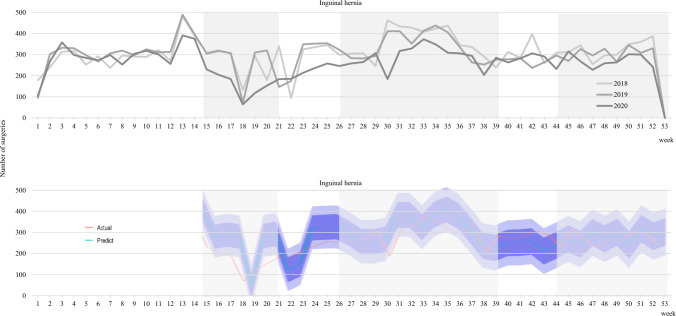
Time-series analysis of weekly changes in the number of inguinal hernia repairs (2018–2020). A model was created to predict the number of surgeries based on pre-pandemic data, and the number of post-pandemic surgeries predicted by the model was plotted over the actual number of surgeries. The 70% and 95% confidence intervals for the predicted number of surgeries are also displayed

The total number of pediatric surgeries recorded in 2018, 2019, and 2020 were 50,026, 49,794, and 45,621. Thus, there was an 8.8% decrease in 2020 from 2018 and an 8.4% decrease in 2020 from 2019 (Table 1). Specific procedures with a > 10% decrease between 2018 and 2020 were inguinal hernia repair (16%), funnel chest surgery (21%), and fundoplication (10%) at disease level A; lung resection (19%) and congenital biliary dilatation surgery (28%) at disease level B; and gastrointestinal atresia surgery (22%) and primary repair of gastroschisis (33%) at disease level C (Table 2). However, a < 10% decrease was observed for orchidopexy (6%), colostomy closure (2%), Hirschsprung’s disease radical surgery (5%), and excision of mediastinal, retroperitoneal, and presacral benign tumors (3%) at disease level A; and appendectomy (3%), gastrointestinal perforation surgery (7%), low-level anorectal malformation radical surgery (6%), and malrotation surgery (9%), at disease level C (Table 2). There was an increase in the number of cases between 2018 and 2020 for gastrostomy (5%) at disease level A; gastrointestinal obstruction surgery (5%) and high–mid-level anorectal malformation radical surgery (7%) at disease level B; and malignant tumor radical surgery (1%) and diaphragmatic hernia repair (6%) at disease level C (Table 2). On evaluating the monthly changes in the number of advanced surgeries by region, we noted that in regions with high infection rates, there was a sharp decline during the first wave, which continued even when an increase in advanced surgeries was expected in July and August, leading to a further decline in the number of advanced surgeries in the second wave. The number of advanced surgeries started increasing from the middle of the third wave. In areas with medium infection levels, there was no change at first, and the number remained unchanged when an increase was predicted in July and August; however, there has been a decline since the third wave. In areas with low infection levels, no noticeable phenomena were observed, with the number of advanced surgeries remaining within the expected range (Fig. 1a–c). Regarding weekly changes in the number of inguinal hernia repairs, a decrease lower than the predicted value was observed early in the first wave, which continued until just before the second wave. Thereafter, a decrease was noted later in the second wave. In the third wave, the number returned to the predicted value and remained there. (Fig. 2). Regarding the severity of appendicitis, there was a difference in severity (*p* = 0.030) and drain insertion (*p* = 0.010) (Table 3). The rates of perforation (*p* = 0.84), abscess formation (*p* = 0.76), and intraperitoneal lavage (*p* = 0.89) were not significant (Table 3). For gastrointestinal obstruction, there was a difference in the operative procedure (*p* = 0.024) (Table 4). The incidence of gastrointestinal obstruction did not decrease, but the rate of adhesiolysis decreased and the rate of bowel resection and enterostomy increased, indicating that the timing of the operation may have been affected by the pandemic (Table 4). There was no difference in the number of emergency transports, but the number of emergency surgeries differed significantly (*p* < 0.001) (Table 5). On evaluating postoperative outcomes, there was a difference in the reoperation rate within 30 days after surgery (*p* = 0.014) but no difference in the postoperative readmission or mortality rates (Table 6).

**Table 1:** Number of operations performed in the regional infection level classification compared with 2018

Infection level	Year	*n*	95% CI		Compared with 2018
			Lower	Upper	
Total	2018 2019 2020	50,026 49,794 45,621			
High	2018	29,971	29,631.7	30,310.3	1.00
	2019	30,005	29,665.5	30,344.5	1.00
	2020	27,336	27,011.9	27,660.1	0.91
Medium	2018	15,080	14,839.3	15,320.7	1.00
	2019	14,892	14,652.8	15,131.2	0.99
	2020	13,980	13,748.3	14,211.7	0.93
Low	2018	4975	4836.8	5113.2	1.00
	2019	4897	4759.8	5034.2	0.98
	2020	4305	4176.4	4433.6	0.87

**Table 2:** Number of operations performed for each procedure in disease level by surgical triage compared with 2018

Disease level	Procedure	Year	*n*	95% CI		Compared with 2018
				Lower	Upper	
Level A	Inguinal hernia repair	2018 2019 2020	16,273 15,958 13,690	16,023.0 15,710.4 13,460.7	16,523.0 16,205.6 13,919.3	1.00 0.98 0.84
	Orchidopexy	2018 2019 2020	4525 4296 4253	4393.2 4167.5 4125.2	4656.8 4424.5 4380.8	1.00 0.95 0.94
	Gastrostomy	2018 2019 2020	836 757 877	779.3 703.1 819.0	892.7 810.9 935.0	1.00 0.91 1.05
	Colostomy closure	2018 2019 2020	500 534 490	456.2 488.7 446.6	543.8 579.3 533.4	1.00 1.07 0.98
	Fundoplication	2018 2019 2020	436 374 392	395.1 336.1 353.2	476.9 411.9 430.8	1.00 0.86 0.90
	Funnel chest surgery	2018 2019 2020	326 306 257	290.6 271.7 225.6	361.4 340.3 288.4	1.00 0.94 0.79
	Hirschsprung’s disease radical surgery	2018 2019 2020	243 227 231	212.4 197.5 201.2	273.6 256.5 260.8	1.00 0.93 0.95
	Excision of mediastinal, retroperitoneal, and presacral benign tumors	2018 2019 2020	180 206 174	153.7 177.9 148.1	206.3 234.1 199.9	1.00 1.14 0.97
Level B	Gastrointestinal obstruction surgery	2018 2019 2020	258 295 270	226.5 261.3 237.8	289.5 328.7 302.2	1.00 1.14 1.05
	High–mid-level anorectal malformation radical surgery	2018 2019 2020	180 198 192	153.7 170.4 164.8	206.3 225.6 219.2	1.00 1.10 1.07
	Congenital biliary dilatation surgery	2018 2019 2020	222 224 160	192.8 194.7 135.2	251.2 253.3 184.8	1.00 1.01 0.72
	Lung resection	2018 2019 2020	166 145 134	140.7 121.4 111.3	191.3 168.6 156.7	1.00 0.87 0.81
Level C	Appendectomy	2018 2019 2020	4463 4567 4349	4332.1 4593.9 4219.7	4593.9 4699.5 4478.3	1.00 1.02 0.97
	Gastrointestinal perforation surgery	2018 2019 2020	491 464 459	447.6 421.8 417.0	534.4 506.2 501.0	1.00 0.95 0.93
	Malignant tumor radical surgery	2018 2019 2020	322 341 324	286.8 304.8 288.7	357.2 377.2 359.3	1.00 1.08 1.01
	Low-level anorectal malformation radical surgery	2018 2019 2020	325 280 304	289.7 247.2 269.8	360.3 312.8 338.2	1.00 0.86 0.94
	Gastrointestinal atresia surgery	2018 2019 2020	367 300 288	329.5 266.1 254.7	404.5 333.9 321.3	1.00 0.82 0.78
	Malrotation surgery	2018 2019 2020	237 247 216	206.8 216.2 187.2	267.2 277.8 244.8	1.00 1.04 0.91
	Diaphragmatic hernia repair	2018 2019 2020	195 206 207	167.6 177.9 178.8	222.4 234.1 235.2	1.00 1.06 1.06
	Primary repair of gastroschisis	2018 2019 2020	193 171 129	165.8 145.4 106.7	220.2 196.6 151.3	1.00 0.89 0.67

**Table 3:** Severity of appendicitis and operative findings

Appendicitis	2018, *N* = 4463	2019, *N* = 4567	2020, *N* = 4349	*p* value
The severity, *n* (%)				0.030
Gangrenous	2039 (46)	2035 (45)	2007 (46)	
Phlegmonous	1321 (30)	1359 (30)	1249 (29)	
Catarrhal	618 (14)	715 (16)	595 (14)	
Normal	485 (11)	458 (10)	498 (11)	
Operative findings, perforation, *n* (%)				0.84
Perforated	713 (16)	711 (16)	694 (16)	
No perforation	3750 (84)	3856 (84)	3655 (84)	
Operative findings, abscess, *n* (%)				0.76
Abscess formation	771 (17)	806 (18)	777 (18)	
No abscess formation	3692 (83)	3761 (82)	3572 (82)	
Operative findings, intraperitoneal lavage, *n* (%)				0.89
With intraperitoneal lavage	2138 (48)	2188 (48)	2064 (47)	
No intraperitoneal lavage	2325 (52)	2379 (52)	2285 (53)	
Operative findings, drain insertion, *n* (%)				0.010
With drain insertion	452 (10)	410 (9.0)	360 (8.3)	
No drain insertion	4011 (90)	4157 (91)	3989 (92)

**Table 4:** Degree of contamination and the operative procedures for gastrointestinal obstruction

Gastrointestinal obstruction	2018, *N* = 258	2019, *N* = 295	2020, *N* = 270	*p* value
The degree of contamination, *n* (%)				0.65
Infected	3 (1.2)	3 (1.0)	3 (1.1)	
Contaminated	17 (6.6)	16 (5.4)	14 (5.2)	
Semi-clean	133 (52)	156 (53)	160 (59)	
Clean	105 (41)	120 (41)	93 (34)	
Operative procedures, *n* (%)				0.024
Adhesiolysis	210 (81)	245 (83)	207 (77)	
Bowel resection	46 (18)	40 (14)	49 (18)	
Enterostomy	2 (0.8)	10 (3.4)	14 (5.2)

**Table 5:** The status of emergency response

	2018, *N* = 50,026	2019, *N* = 49,794	2020, *N* = 45,621	*p* value
Emergency transport, *n* (%)				0.78
With transport	2876 (5.7)	2839 (5.7)	2649 (5.8)	
No transport	47,150 (94.3)	46,955 (94.3)	42,972 (94.2)	
Emergency surgery, *n* (%)				< 0.001
Emergency surgery	8912 (17.8)	9112 (18.3)	8589 (18.8)	
Non-emergency surgery	41,109 (82.2)	40,677 (81.7)	37,028 (81.2)	
Unknown	5	5	4

**Table 6:** Outcomes and mortality rates

	2018, *N* = 50,026	2019, *N* = 49,794	2020, *N* = 45,621	*p* value
Unexpected reoperation within 30 days after surgery, *n* (%)				0.014
With reoperation	619 (1.2)	640 (1.3)	659 (1.4)	
No reoperation	49,398 (98.8)	49,148 (98.7)	44,954 (98.6)	
Unknown	9	6	8	
Unplanned readmission within 30 days after surgery, *n* (%)				0.90
With reoperation	337 (0.7)	335 (0.7)	317 (0.7)	
No reoperation	49,678 (99.3)	49,443 (99.3)	45,295(99.3)	
Unknown	11	16	9	
Mortality, *n* (%)				0.19
Operative	155 (0.3)	193 (0.4)	144 (0.3)	
Survival	49,548 (99.0)	49,299 (99.0)	45,197 (99.1)	
Non-operative	323 (0.6)	302 (0.6)	280 (0.6)	

## Discussion

During the COVID-19 pandemic, hospitals were forced to curtail elective surgeries to prevent infection spread [2, 3]. To conserve resources and avoid the risk of infection, urgent surgeries and emergency transport were restricted and treatments were prioritized as urgent, emergency, or elective depending on the risk associated with their delay (life-threat, harm, or negligible, respectively) [10]. We analyzed the impact of the COVID-19 pandemic on pediatric surgeries in Japan based on various parameters such as disease, region according to infection status, and time. The NCD data used for the analysis are from one of the highest-quality databases containing most of the surgical data collected by surgeons throughout Japan, ensuring complete coverage and accuracy. Using this nationwide dataset, we compared data before and during the COVID-19 pandemic and examined its impact on pediatric surgical treatment. Although the total number of operations decreased by 8.8% and 8.4% in 2020, the annual decrease in the number of patients aged < 15 years (15.41, 15.21, and 15.12 million in 2018, 2019, and 2020, respectively) [11–13] during the same period ranged from 1.3% to 0.6%, suggesting that the decrease in 2020 exceeded that of the population, and that the decline in 2020 had a greater impact than the decline in the population.

### Regional infection level

A decrease in the number of surgeries according to the prevalence of infection in an area was observed in all regions. Interestingly, the impact of coronavirus infection was greater in areas with low infection rates than in areas with high infection rates, although the impact of regional changes in resource retention and emergency transport remains unknown. In regions with a high number of infected patients, a decrease in the number of cases of highly advanced surgeries was observed during the summer vacation period (second wave) and in October (third wave), following the first wave in April, 2020. Because the infection spread quickly, regions with high infection levels tended to experience a rapid decrease in the number of highly advanced surgeries, with the number of surgeries increasing quickly thereafter. In regions with medium–low infection levels, the numbers of highly advanced surgeries decreased gradually because of the limitation in medical resources as the pandemic continued.

### Surgical triage

In terms of changes in disease triage levels, the number of cases of inguinal hernia and funnel chest surgeries declined for disease level A, whereas the number of cases of gastrostomy increased. The decrease in the number of inguinal hernia repairs, which are often treated by scheduled surgery, may be the result of restrictions to prevent infection or a decrease in opportunities for pediatric consultation and checkups, given that there were few cases of COVID-19 reported in children in 2020. The increase in gastrostomy procedures may have increased their significance as palliative surgery. Funnel chest has a relatively long observation period before a surgical decision is made and, in some cases, the timing of surgery is considered on a yearly basis; therefore, it is expected that surgery would be avoided during the pandemic. For disease level B, there was a > 10% reduction in the number of surgeries for lung resection and congenital biliary dilatation. Similarly, for patients with relatively stable symptoms who had been observed for long periods, surgery during the COVID-19 pandemic was avoided and conservative follow-up was initiated. For disease level C, the number of surgeries for gastrointestinal atresia and primary repair of gastroschisis decreased by > 10%. It is possible that radical surgery was avoided and palliative surgery was selected, but a detailed examination of neonatal cases is necessary to confirm this. Variations by disease were seen, but there were no trends by disease triage level.

During large-scale earthquakes, disasters, and typhoons, triage in emergency medical care is conducted at the disaster site and evacuation centers. However, during the COVID-19 pandemic, it was unknown whether triage was conducted in general medical care settings because of the possibility of transmission of infection. In this study, we examined the impact of triage on the number of surgeries based on common triage methods and found that the number of surgeries varied according to disease specificity, regional characteristics, infection status, season, and other factors.

### Appendicitis

Diseases that are expected to increase in severity and mortality as a result of changes in the patient’s condition include appendicitis, intestinal obstruction, gastrointestinal perforation, and intestinal rotation abnormalities. There have been reports of an increase in the incidence of appendicitis during the COVID-19 epidemic [14]. Appendicitis management during the pandemic resulted in the suspension of elective schedules to preserve resources [15]. In some institutions, uncomplicated appendicitis was managed nonsurgically, or deferred interval appendectomy was considered [15, 16]. By selecting treatments based on the severity of the condition, it was possible to reduce the impact of the delay on treatment outcomes. Based on the present results, no progression or worsening of inflammatory changes were observed in histological and intraoperative findings, and it is considered that the patients’ condition did not worsen if they refrained from visiting a hospital or the time to diagnosis was delayed. In the present study, the number of cases for which conservative treatment was performed and standby appendectomy was selected is unknown. For intestinal obstruction, gastrointestinal perforation, and malrotation, there was no evidence of progression such as in the incidence of contamination; however, the number of cases of intestinal resection and short bowel syndrome increased. There was no effect to change the severity of these diseases.

### Emergency medical system

A significant decrease in the number of emergency room visits during the COVID-19 pandemic was reported worldwide, with over-utilization during normal times and withholding of visits during infectious periods cited as reasons [17]. There was no change in the number of emergency transportations. Knowledge of the emergency procedures in the infected area may clarify this. However, since the NCD data is based on the number of surgical procedures, it was not possible to confirm the local medical delivery system. Regarding postoperative results, the reoperation rate within 30 days after surgery increased, but the readmission rate and mortality rate did not change. Moreover, gastrointestinal perforation, which has the highest mortality rate among neonatal diseases, decreased to 11.2%, 11.9%, and 9.6% in 2018, 2019, and 2020, respectively. While the mortality rate associated with neonatal diseases has been declining year by year, there has been no improvement in the outcome of gastrointestinal perforation [18, 19]; therefore, the factors behind the mortality rate are of interest.

### Limitations

Our study has several limitations. First, it included NCD data from institutions where pediatric surgeries are performed on an ongoing basis. While most neonatal to infant surgeries and highly advanced surgeries are covered, appendicitis may not have been registered as these surgeries are also performed as general adult surgery. However, the number of facilities entering the NCD data in the field of pediatric surgery is constant from year to year, ensuring complete coverage and accuracy of the results; thus, we believe that the findings are reliable for future comparisons. Second, the participating hospitals in this study varied among university hospitals, children's hospitals, and general hospitals, and the acceptance of patients with COVID-19 may have differed among these facilities. Since infection control systems and medical resources were generalized nationwide early, we believe that this selection bias is minimal and that these results are important for understanding the course of the study in the future. Third, there were relatively few pediatric COVID-19-positive cases during the study period. Notably, the decrease in the number of surgeries is expected to be related to prophylaxis, which is a secondary effect. Moreover, predicting whether these results can be applied elsewhere is difficult since there is a considerable difference between the national prevalence of COVID-19 and the situation worldwide. Thus, further global analyses are warranted. Finally, regarding statistical analysis, the predictive performance of the prediction model with pre-pandemic data is limited because only 2 years of pre-pandemic data were available.

## Conclusion

Our analysis of the NCD data may reveal the impact of COVID-19 on pediatric surgery objectively. In 2020, the number of surgeries decreased, but most of these were scheduled surgeries, whereas there was no decrease in the number of emergency surgeries. Moreover, the risk of severe or potentially fatal disease did not decrease or worsen, and the mortality rate of these diseases did not change significantly, which is considered to be the result of resource allocation, surgical triage, and emergency systems responding according to the infection level in each region. This is expected to have a further impact in the context of the ongoing spread of COVID-19 infection. The effects of unknown infectious diseases will require adjustments to the healthcare environment in the future.

## References

[1] Zhu N, Zhang D, Wang W, Li X, Yang B, Song J, et al. A novel coronavirus from patients with pneumonia in China. N Eng J Med. 2019;382:727–33. 10.1056/NEJMoa2001017 PMC7092803 31978945

[2] COVID Surg Collaborative. Elective surgery cancellations due to the COVID-19 pandemic: global predictive modeling to inform surgical recovery plans: elective surgery during the SARS-CoV-2 pandemic. Br J Surg. 2020;107:1440–9. 32395848 10.1002/bjs.11746 PMC7272903

[3] COVID Surg Collaborative. Global guidance for surgical care during the COVID-19 pandemic. Br J Surg. 2020;107:1097–103. 32293715 10.1002/bjs.11646 PMC7262310

[4] Klazura G, Park P, Yap A, Laverde R, Bryce E, Cheung M, et al. Pediatric surgical wait list in low middle income countries during the COVID-19 pandemic. J Surg Res. 2023;288:193–201. 37018896 10.1016/j.jss.2023.02.012 PMC9970937

[5] National Institute of Infectious Diseases. Epidemiological study of gender and age characteristics of each epidemic wave of novel coronavirus infection in Japan. Infectious Agents Surveillance Report. 2022; 43:273–5. https://www.niid.go.jp/niid/ja/typhi-m/iasr-reference/2605-related-articles/related-articles-514/11696-514r01.html. Accessed 12 Dec 2022. **(in Japanese)**

[6] Japanese Society of Pediatric Surgeons, Committee for Academic and Advanced Medical Care. National Clinical Database, Pediatric Surgery. Annual Report 2019. Nissyougekaisi. (J Jpn Pediatr Surg). 2021; 57: 889–895. **(in Japanese)**

[7] Recommendations for Surgical Procedures for Patients with Positive or Suspected New Coronavirus, Japanese Society of Pediatric Surgeons. http://www.jsps.or.jp/coronavirus-19. Accessed 12 Dec 2022. **(in Japanese)**

[8] Ikeda N, Yamamoto H, Taketomi A, Hibi T, Ono M, Niikura N, et al. The impact of COVID-19 on surgical procedures in Japan: analysis of data from the National Clinical Database. Surg Today. 2022;52:22–35. 34783905 10.1007/s00595-021-02406-2 PMC8592826

[9] R Core Team. R: a language and environment for statistical computing. R Foundation for Statistical Computing; 2021. https://www.R-project.org/. Accessed 21 July 2023.

[10] Dedeilia A, Esagian S, Ziogas I, Giannis D, Katsaros I, Tsoulfas G. Pediatric surgery during the COVID-19 pandemic. World J Clin Pediatr. 2020;9:7–16. 33014718 10.5409/wjcp.v9.i2.7 PMC7515751

[11] Population estimate. Statistics Bureau of Japan; 2019. https://www.stat.go.jp/data/jinsui/2018np/index.html. Accessed 12 Dec 2022. **(in Japanese)**.

[12] Population estimate. Statistics Bureau of Japan; 2020. https://www.stat.go.jp/data/jinsui/2019np/index.html. Accessed 12 Dec 2022. **(in Japanese)**.

[13] Population estimate. Statistics Bureau of Japan; 2021. https://www.stat.go.jp/data/jinsui/topics/topi1251.html. Accessed 12 Dec 2022. **(in Japanese)**.

[14] Motazedian G, Aryanpoor P, Rahmanian E, Abiri S, Kalana N, Hatami N, et al. Incidence of pediatric perforated appendicitis during the COVID-19 pandemic a systematic review and meta-analysis. Arch Acad Emerg Med. 2022;10:3. 10.22037/aaem.v10i1.1421 PMC8771157 35072092

[15] Polites S, Azarow K. Perspectives on pediatric appendicitis and appendectomy during the severe acute respiratory syndrome coronavirus 2 pandemic. J Laparoendosc Adv Surg Tech A. 2020;30:356–7. 32233967 10.1089/lap.2020.0197

[16] Huang L, Yin Y, Yang L, Wang C, Li Y, Zhou Z. Com-parison of antibiotic therapy and appendectomy for acute uncomplicated appendicitis in children a meta-analysis. JAMA Pediatr. 2017;171:426–34. 28346589 10.1001/jamapediatrics.2017.0057 PMC5470362

[17] Ojetti V, Covino M, Brigida M, Petruzziello C, Saviano A, Migneco A, et al. Non-COVID diseases during the pandemic where have all other emergencies gone? Medicia (Kaunas). 2020;56:512. 10.3390/medicina56100512 PMC7599851 33019514

[18] Yagi M, Kohno M, Asagiri K, Ikeda T, Kanada S, Kawashima S, et al. Twenty-year trends in neonatal surgery based on a nationwide Japanese surveillance program. Pediatr Surg Int. 2015;31:955–62. 26319695 10.1007/s00383-015-3775-z

[19] Japanese Society of Pediatric Surgeons, Committee for Academic and Advanced Medical Care. Current status of neonatal surgery in our country—national Tally of Neonatal Surgery in Japan in 2018. Nissyougekaisi. (J Jpn Pediatr Surg). 2020; 56:1167–82. **(in Japanese)**.

